# Mercy Hospital

**Published:** 1870-03

**Authors:** 


### MERCY HOSPITAL.

#### CLINIC OF N. S. DAVIS, M.D., PROF. OF CLINICAL MEDICINE, ETC.

Hydrocephalus.—The first case brought to the attention of the class, at a recent clinic, was a male child, 21 months old, presenting all the symptoms of chronic hydrocephalus, such as marked enlargement of the head, open condition of the fontanelles, weakness of the lower extremities, inability to stand on its feet, etc.

The mother stated that the child appeared healthy and well formed, until between four and six months old, when it was attacked with fever, accompanied by much heat in the head, hurried breathing, frequent and sudden startings, screeching, and restlessness, but no general convulsions. These acute symptoms continued nearly two weeks, when the fever gradually declined, leaving the child, however, restless, and subject to starting and crying out in its sleep; its urine scanty; intestinal discharges often unnatural, and subject to occasional vomiting. In a few weeks its head was found to have increased in size, much out of proportion to the body, and the anterior fontanella more convex or full than natural. Its attention could not be fixed upon any object, and it made no attempt to stand on its feet when lifted to an upright position; although, when recumbent, it would move its lower extremities freely, and its tissues were generally well nourished. In this condition, the child was first brought to the hospital, three weeks since, and the class allowed to examine its condition and symptoms in detail. The diagnosis, so far as determining the existence of a considerable amount of serous effusion into the ventricles and on the surface of the brain, was easy; but whether such effusion was the result of simple, primary meningitis, degenerating into a chronic form, or whether it was accompanied by and dependant on tubercular deposits, in the arachnoid and pia-mater, was a question of more difficult solution.

From the absence of any tuberculous condition of the parents and the generally good condition of nutrition in the child, the opinion was expressed that the case was not complicated with tuberculosis. The indications for treatment were to leave the child comparatively quiet, feed it only fluid and easily assimilated food, carry it out in the open air when the weather is fair, and give it such medicine as is calculated to allay irritation in the cerebral membranes, and so far promote the action of the kidneys as to induce the gradual absorption of the fluid effused into the cavity of the cranium. To accomplish these purposes, the child, when first brought to the hospital, three weeks since, was put on the use of the following prescription:—

℞. Fl. Ext. Scutellaria, ---------------------- ℥ij.
Tinct. Digitalis, ---------------------------- ℥ss.
Iodide Potassa, ----------------------------- ʒij.

Mix, and give 15 drops four times a day in sweetened water.

The mother had carried out the directions faithfully, and a favorable change is easily recognized. The quantity of urine has been much increased, the sleep is more quiet, the child more cheerful and disposed to notice playthings, the expression of countenance more intelligent, and the fontanella less prominent and perceptibly smaller. These changes afford just reason for hopes of a final recovery. The same treatment was continued.

Muscular and Bronchial Rheumatism. — This was a case of a laboring man, 21 years of age, admitted into the hospital about two weeks since, complaining of pain and soreness in the chest, frequent, harsh cough, with but little expectoration, and moderate fever. There were no physical signs of pneumonia; and the case was regarded as one of catarrhal irritation of the bronchial tubes, coupled with rheumatic soreness in the muscles of the chest and shoulders.

He was directed to take one teaspoonful of the following prescription every four hours:

℞. Hydrochlorate of Ammonia,------------- ʒiij.

Tart. Ant. et Pot.,---------------------- 2 grs.

Sulph. Morph.,-------------------------- 3 grs.

Syrup Liquorice,------------------------ ℥iv.

Mix.

Under the influence of this, the cough ceased and the pains in the chest diminished; but in three or four days he began to complain of nausea, great weakness and soreness in the muscles of the thighs and calves of the legs, aggravated by motion. The rhythm of the heart was natural, but the first sound was muffled or gruff, and the impulse increased.

The former prescription was omitted and the two following given instead:—

℞. Hydrochlorate of Ammonia, -------------- ʒiij.

Tinct. Aconite Root,---------------------- ʒj.

Syrup Liquorice,-------------------------- ℥iv.

Mix. Give one teaspooful every six hours, in a little water.

℞. Bi-Carb. Soda,------------------------------- ʒij.

Sub-Nit. Bismuth, ------------------------- ʒij.

Mix. Divide 20 powders; one every six hours, alternately, with the other prescription; animal broth and milk for nourishment.

This treatment was continued three or four days, during which the cardiac sounds and impulse became more natural, the pulse slower and more soft, and the muscular soreness in the lower extremities less. But the countenance of the patient was depressed and anxious; the skin wet with a cold, stickey sweat; the tongue dry and red along the middle of the upper surface; and his bowels slightly relaxed, as though there was a typhoid tendency.

He was put on the use of a powder composed of sulph. quinine, 2 grs., and sub. nit. bismuth, 5 grs., each morning, noon, tea-time, and bed-time.

A decided improvement was observable in the condition of the skin, tongue, and countenance, the next day. Four days have since elapsed, and the improvement has continued, until at present, the patient appears to be convalescent. The Prof. spoke of several other cases of well-marked rheumatic fever, which had lately been under his care, in which, under the free use of alkaline salts, the rheumatic symptoms abated, but the tongue became dry in the middle, the skin cool and clammy, pulse quick and weak, with disgust for food and mental despondency, in which the combination of bismuth and quinine produced a rapid improvement, until convalescence was established.

Prurigo.—Mr. L., aged 25 years, had just been admitted into the hospital. His general health appeared good, but he had been for a considerable time excessively annoyed with an itching eruption on the skin. For three or four years it had made its appearance with the cold and wet weather of autumn, continued through the winter, and disappeared with the coming of warm weather in the spring. It shows most on the nates, inner and posterior part of the thighs, legs, and forearms. It is in the form of small, scattered elevations in the skin, only slightly reder than natural, and with no vescicle, or tendency to suppurate.

In other words, it consists of small, isolated papules; many of them having thin, black scabs on them, produced by the patient having torn them by scratching, until the blood started. The itching in these cases is entirely disproportioned to the external appearances of disease.

It is increased by warmth, and is generally worst when the patient gets warm in bed; often making the night as restless as if the bed had been sprinkled with cowage or nettles. The fact that the disease makes its appearance in this patient uniformly in the autumn shows that it is dependant on the retention of some effete matter, that ought to be eliminated through the skin, but which is prevented by the influence of cold and damp on the surface. The disease is strictly papular, and is usually called prurigo. Cases of this kind are often very persistent, yielding but slowly to the influence of remedies. The treatment most likely to cure the present case, would be such as would first restore a free action of the skin and kidneys, until all retained effete matter had been eliminated; and then, perhaps, a cautious use of some arsenical preparation, for two or three weeks. The class were cautioned against the use of the latter preparations, empirically, for such cases, as they were capable of doing much mischief. This patient had, indeed, been using sulphur vapor-baths and liquor arsenicalis, until symptoms of œdema had supervened, before he came to the hospital; and yet without benefit. He was directed to take a warm alkaline bath every second day, and to apply to the itching surface the following ointment every night:—

℞. Iodide Potass, ---------------------------- ʒj.

Simple Cerate, ---------------------------- ℥iij.

Mix thoroughly.

Internally he was directed the following:—

℞. Vin. Colchici Sem., ------------------------ ℥j.

Acetis Potass, ---------------------------- ℥ss.

Syrupi Simplex, ---------------------------- ℥j.

Aqua, ------------------------------------ ℥ij.

Mix. Take a teaspoonful four times a day. Should the colchicum affect the bowels too much, half a drachm of camphorated tincture of opium can be added to each dose.

If after the ointment of iodide potassa has been used four or five nights, it is found to create too much irritation in the skin, as it sometimes does, it may be omitted and the following wash substituted in its place:—

℞. Bi-chloride Hydrg, ---------------------- 10 grs.
Aqua Camph., ---------------------- ℥viij.
Glycerine, ---------------------- ℥ij.

Mix. Apply as a wash every night and morning. In most cases like this, the foregoing treatment will afford relief, in from ten days to two weeks.

